# Microarray-based analysis of fish egg quality after natural or controlled ovulation

**DOI:** 10.1186/1471-2164-8-55

**Published:** 2007-02-21

**Authors:** Emilie Bonnet, Alexis Fostier, Julien Bobe

**Affiliations:** 1 INRA, UR1037 SCRIBE, IFR140, Ouest-Genopole, 35000 Rennes, France

## Abstract

**Background:**

The preservation of fish egg quality after ovulation-control protocols is a major issue for the development of specific biotechnological processes (e.g. nuclear transfer). Depending on the species, it is often necessary to control the timing of ovulation or induce the ovulatory process. The hormonal or photoperiodic control of ovulation can induce specific egg quality defects that have been thoroughly studied. In contrast, the impact on the egg transcriptome as a result of these manipulations has received far less attention. Furthermore, the relationship between the mRNA abundance of maternally-inherited mRNAs and the developmental potential of the egg has never benefited from genome-wide studies. Thus, the present study aimed at studying the rainbow trout (*Oncorhynchus mykiss*) egg transcriptome after natural or controlled ovulation using 9152-cDNA microarrays.

**Results:**

The analysis of egg transcriptome after natural or controlled ovulation led to the identification of 26 genes. The expression patterns of 17 of those genes were monitored by real-time PCR. We observed that the control of ovulation by both hormonal induction and photoperiod manipulation induced significant changes in the egg mRNA abundance of specific genes. A dramatic increase of Apolipoprotein C1 (APOC1) and tyrosine protein kinase HCK was observed in the eggs when a hormonal induction of ovulation was performed. In addition, both microarray and real-time PCR analyses showed that prohibitin 2 (PHB2) egg mRNA abundance was negatively correlated with developmental success.

**Conclusion:**

First, we showed, for the first time in fish, that the control of ovulation using either a hormonal induction or a manipulated photoperiod can induce differences in the egg mRNA abundance of specific genes. While the impact of these modifications on subsequent embryonic development is unknown, our observations clearly show that the egg transcriptome is affected by an artificial induction of ovulation.

Second, we showed that the egg mRNA abundance of prohibitin 2 was reflective of the developmental potential of the egg.

Finally, the identity and ontology of identified genes provided significant hints that could result in a better understanding of the mechanisms associated with each type of ovulation control (i.e. hormonal, photoperiodic), and in the identification of conserved mechanisms triggering the loss of egg developmental potential.

## Background

Fish egg quality can be defined as the ability of the egg to be fertilized and subsequently develop into a normal embryo. The egg's potential to produce a viable and normal embryo can be affected by many environmental and biological factors acting at various steps of the oogenetic process (see [1,2] for review). The determinism of egg quality has also been shown to be under the influence of genetic factors [3-5]. While the effects of many experimental factors have been studied, the mechanisms by which they trigger egg quality losses are far less documented. Yolk composition as a result of a specific diet has been intensively studied in several fish species in relationship with egg developmental capacities [6-8]. Hormones of maternal origin supplied to the embryo by the egg also have a significant effect on embryonic development as shown by several studies [9]. In contrast, the putative role of non-yolky cytoplasmic components accumulated during oogenesis, such as structural and regulatory proteins, cortical alveoli content and messenger RNAs (mRNAs), has received far less attention [1]. Nevertheless, maternal mRNAs that accumulate in the oocyte during oogenesis are essential for early embryonic development [10,11]. Like in other animals, some maternal mRNAs are involved in embryonic germ cells formation in fish [12], but other oocyte mRNAs, such as those involved in growth regulation, could be necessary to ensure a normal early development [13]. Thus, in bovine two-cell embryos, a relationship between embryonic developmental competence, assessed in terms of time of first cleavage, and the expression of IGF1 mRNA was reported [14]. In addition, other studies showed a relationship between variation of maternal RNA polyadenylation levels and developmental competence of mammalian oocytes, thus pointing out a relationship between maternal mRNA stability and embryonic developmental capacities [15]. In fish, the possibility that specific oocyte mRNAs might be affected when egg quality is experimentally decreased has been seriously suggested by a previous work dealing with the effect of egg post-ovulatory ageing on the mRNA levels of many genes (~40) in rainbow trout eggs [16].

In fish, it is often useful or necessary to control the timing of spawning or induce the ovulatory process. These techniques are used for biotechnical, experimental or economical reasons to obtain out of season egg production and/or synchronous egg production within a group of females or, for some species, to obtain eggs from captive fish. The effects of these manipulations on fish egg quality have been thoroughly studied [1,17]. However, the impact on egg transcriptome as a result of these manipulations has received far less attention despite recent efforts to study the ovarian or follicular transcriptome during oogenesis [18-20]. In the present study, we analyzed the transcriptome of unfertilized rainbow trout (*Oncorhynchus mykiss*) eggs after natural or controlled ovulation. Two different protocols of controlled ovulation that are widely used in laboratories and fish farms were carried out: (i) a hormonal induction of ovulation using intra-peritoneal GnRH-analog injection, and (ii) a specific photoperiod regime designed to advance the spawning period. In addition, a third group was not subjected to any specific manipulation to allow egg collection after natural spontaneous ovulation. For each individual female, egg samples were collected and either subjected to a microarray analysis or transferred in an experimental hatchery after fertilization for monitoring developmental success (e.g. embryonic survival, malformations). Thus, the present study aimed at (i) analyzing the effect of ovulation control processes on egg transcriptome and (ii) analyzing possible links between egg transcriptome and egg developmental potential.

## Results

### Egg quality

**Figure 1:**
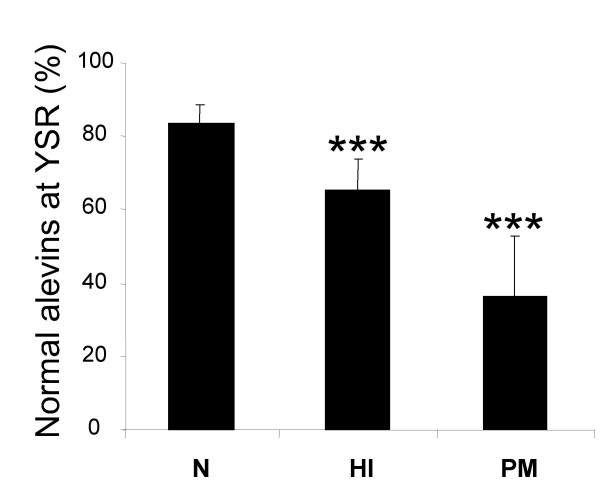
Percentage of normal alevins at yolk sac resorption (mean ± 95% confidence interval) observed after fertilizing eggs of females subjected to natural ovulation (N, n = 25), hormonal induction of ovulation (HI, n = 33) and photoperiod manipulation of ovulation (PM, n = 17). Significantly different from natural ovulation at p < 0.0001 (***).

Both hormonal induction and photoperiodic manipulation of ovulation had a negative impact on egg quality. The percentage of normal (i.e. without morphological abnormalities) alevins monitored at yolk-sac resorption (YSR) was used to characterize the egg quality of each individual female. The higher percentage of normal alevins at YSR, 84 ± 5%, was observed after natural (N) ovulation (Figure 1). In contrast, significantly lower percentages were observed after hormonal induction (HI) of ovulation (65 ± 9%) or photoperiodic manipulation (PM) of ovulation (37 ± 16%) (Figure 1).

### Transcriptomic analysis

**Figure 2:**
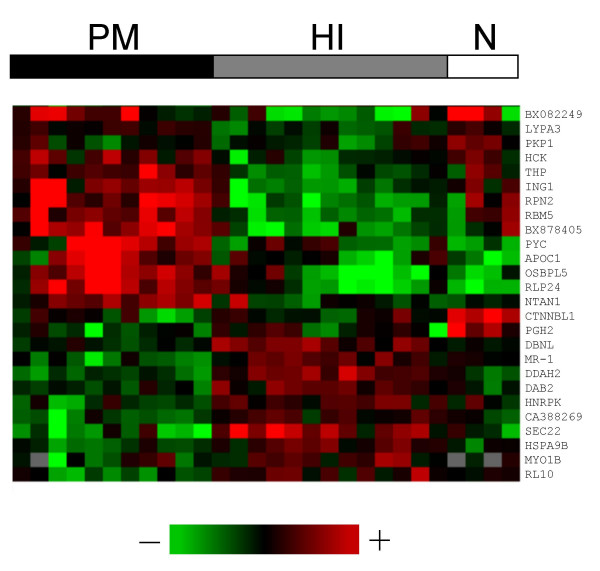
Unsupervised average linkage clustering analysis of the 26 differentially abundant genes in eggs collected after photoperiod-manipulated ovulation (PM), hormonally-induced ovulation (HI) and natural ovulation (N). Each row represents a gene and each column represents an egg RNA sample. For each gene, the expression level within the sample set is indicated using a color intensity scale. Red and green are used for over and under abundance respectively while black is used for median abundance.

**Figure 3:**
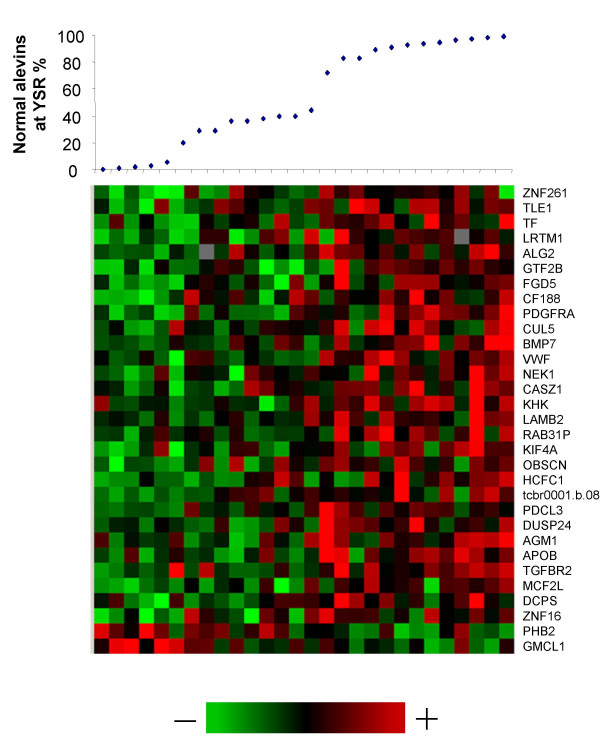
Supervised average linkage clustering analysis of 31 genes significantly linked to egg quality. Each row represents a gene and each column represents an egg RNA sample. The 31 samples are supervised according to the percentage of normal alevins at yolk-sac resorption. For each gene, the expression level within the sample set is indicated using a color intensity scale. Red and green are used for over and under abundance respectively while black is used for median abundance.

After signal processing, 8423 clones out of 9152 were kept for further analysis. SAM analysis was performed using the expression data of those 8423 clones. Twenty six genes exhibiting a differential mRNA abundance among at least 2 of the 3 experimental groups were identified (Table 1, Figure 2) with a false discovery rate (FDR) of 3.4%. The ontologies of those genes are presented in Table 2. Thirty one genes putatively linked to egg quality were identified (Table 3, Figure 3) with a FDR of 30%. The ontologies of those genes are presented in Table 4.

### Real-time PCR analysis

From the 57 (26+31) genes identified in the transcriptomic analysis, 32 were ultimately kept for real-time PCR analysis (Table 5). Real-time PCR data corresponding to the remaining 25 was not used in the analysis because of methodological reasons (e.g. low expression, poor PCR efficiency, double amplification).

#### Genes exhibiting a differential egg mRNA abundance among experimental groups

**Figure 4:**
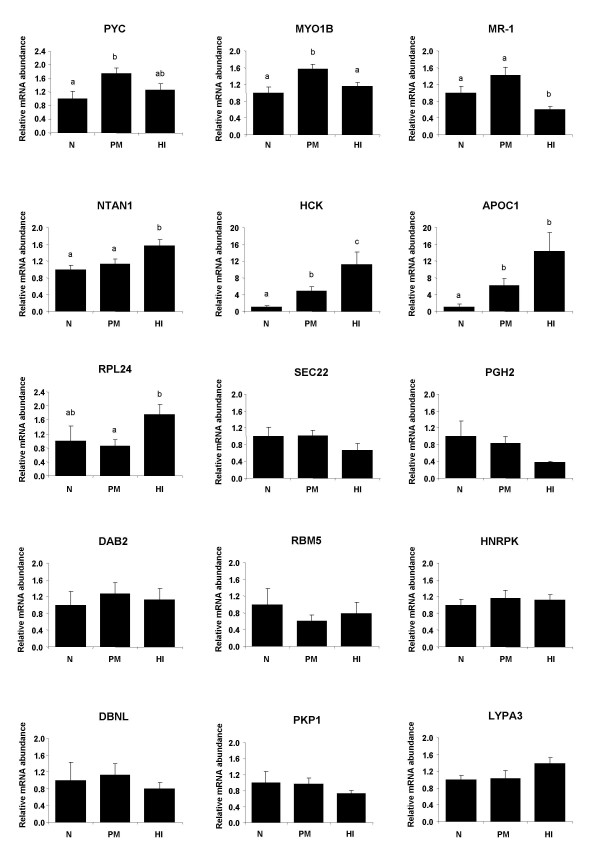
Real-time PCR analysis of gene mRNA abundance (mean ± SEM) in unfertilized eggs collected after natural ovulation (n = 4), hormonally-induced ovulation (n = 11) and photoperiod-manipulated ovulation (n = 14). Different letters indicate significant differences between groups at p < 0.05. The official human symbol is indicated for all studied genes.

Among the 26 genes exhibiting a differential mRNA abundance between experimental groups, 17 were studied by real-time PCR. Among those 17 genes, 7 were found to be differentially expressed in the real-time PCR study (Figure 4). The identity of those 7 genes is presented below. Only the informative alignments obtained using the full rainbow trout coding sequence (CDS) or a substantial part of the CDS are presented (Figures 3, 4). For clarity reasons, the official human protein symbol was used in the text.

**Figure 5:**
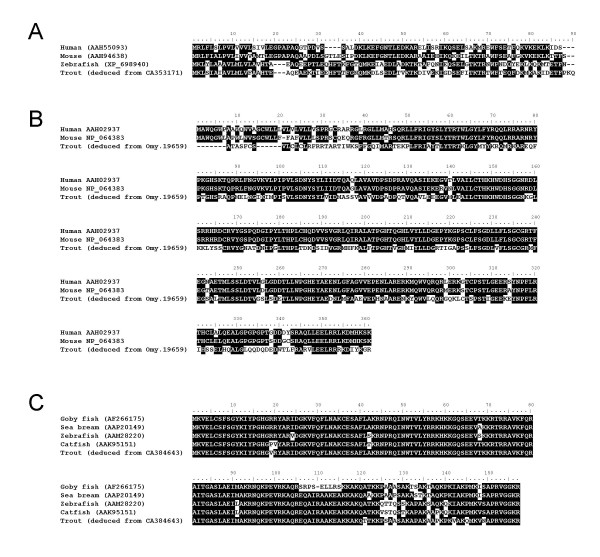
Amino acid sequence alignment of rainbow trout APOC1 (A), MR-1 (B), and RPL24 (C) with cognate vertebrate forms. For each target species, the GenBank accession number of the protein is indicated.

Clone # 1RT65F10_D_C05 exhibited significant sequence similarity with mouse Apolipoprotein C-I precursor (APOC1, Table 1) and was significantly more abundant in eggs of the HI group than in eggs of the N group while intermediate levels were observed in eggs of the PM group. The mRNA abundance in the HI group was 13 times higher than in the N group while it was 2 times higher than in the PM group (Figure 4). After performing a Blast search in the GenBank database, the complete rainbow trout amino acid sequence deduced from the EST sequence exhibited 54% sequence identity at the amino acid level with the zebrafish (*Danio rerio*) cognate protein (Figure 5A). A sequence identity of 33 and 26% was observed with mouse and human proteins respectively (Figure 5A). The number of amino acids deduced from the trout EST is consistent with the number of amino acids present in mammalian and zebrafish sequences.

A similar expression pattern was observed for clone # 1RT68D18_D_B09 that exhibited sequence similarity with mouse Hemopoietic cell kinase (HCK, Table 1). The deduced partial amino acid sequence generated from the corresponding UniGene cluster exhibited 40% and 38% identity with mouse and human HCK proteins respectively.

Clone tcbk0023.o.24 exhibited sequence similarity with hydroxyacylglutathione hydrolase cytoplasmic (MR-1, Table 1) and was less abundant in eggs of the HI group than in eggs of the 2 other experimental groups (Figure 4). A contig sequence was generated using all rainbow trout ESTs belonging to the same UniGene cluster (Omy.19659). This contig sequence was then used to perform a blastX search in GenBank. This contig sequence corresponded to a partial CDS of the putative rainbow trout cDNA. The deduced rainbow trout amino acid sequence exhibited 59% identity with the mouse brain protein 17 isoform 1 (Figure 5B). This mouse protein is also known as myofibrillogenesis regulator 1. In addition a 60% identity was observed with human cognate protein (Figure 5B) know as myofibrillogenesis regulator 1 (MR-1).

Clone tcba0025.n.15 exhibited sequence similarity with human N-terminal asparagine amidase (NTAN1, Table 1) and was more abundant in eggs of the HI group than in eggs of N and PM groups (Figure 4). This sequence did not belong to any UniGene cluster and did not include a complete CDS. After performing a Blast search using this partial sequence, a 47% identity with the cognate human form (NTAN1) was observed.

Clone 1RT131K20_C_F10) exhibited sequence similarity with mouse myosin Ib (MYO1B, Table 1) and was more abundant in eggs of the PM group than in eggs of HI and N groups (Figure 4). This sequence did not belong to a UniGene cluster and did not contain a full CDS. The observed identity with predicted zebrafish and chicken cognate forms was 93 and 86% respectively. An 85 and 86% amino acid sequence identity was observed with human and murine proteins respectively.

Clone tcay0027.b.13 exhibited sequence similarity with human pyruvate carboxylase (PYC, Table 1) and was more abundant in eggs of the PM group than in eggs of the N group, while intermediate levels were observed in eggs of the HI group (Figure 4). This sequence did not include a full CDS. After performing a Blast search using this partial coding sequence, the amino acid sequence identity with cognate vertebrate forms was above 80%.

Clone 1RT139F11_B_C06 exhibited sequence similarity with ribosomal protein RPL24 and was more abundant in eggs of the HI group than in eggs of the PM group (Figure 4). This clone included a full CDS and the deduced amino acid sequence exhibited very strong (above 95%) sequence identity with cognate fish proteins (Figure 5C).

For 5 genes (HRNPK, RBM5, DAB2, PGH2 and SEC22, Table 1) similar expression profiles were observed in real-time PCR and microarray analyses. However, no statistical differences between groups were observed in the real-time PCR experiment (Figure 4).

For 3 genes (PKP1, DBNL and LYPA3, Table 1) the consistency between real-time PCR and microarray data was limited to 2 of the 3 experimental groups. In addition, no statistical differences between groups were observed in the real-time PCR analysis (Figure 4).

For the 2 remaining clones (BX082249 and CA388269, Table 1), no correlation was observed between real-time PCR and microarray data (data not shown).

#### Genes exhibiting a quality-dependent mRNA abundance in the eggs

**Figure 6:**
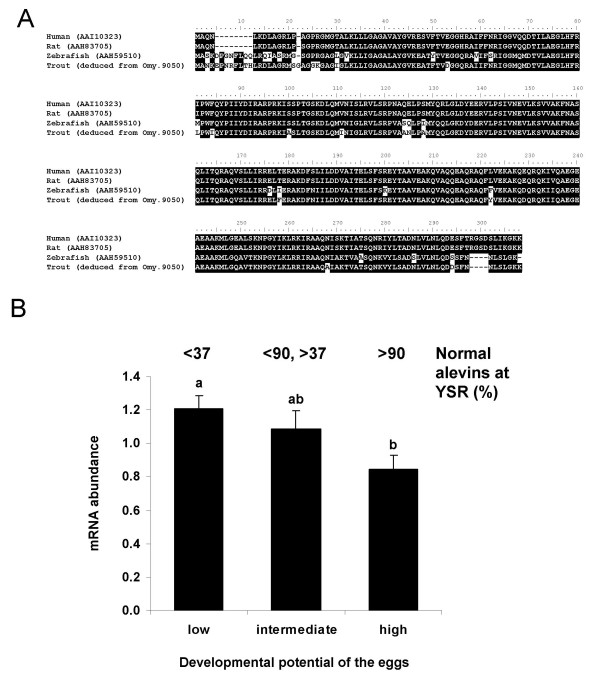
(A) Amino acid sequence alignment of deduced rainbow trout prohibitin 2 (PHB2) with human, rat and zebrafish forms. For each target species, the GenBank accession number of the protein is indicated. (B) Real-time PCR analysis of PHB2 mRNA abundance (mean ± SEM) in eggs of low (n = 10), intermediate (n = 9) and high (n = 10) developmental potential estimated by the percentage normal alevins at yolk-sac resorption. Different letters indicate significant differences between groups at p < 0.05.

Among the 31 genes identified as linked to egg quality, 15 were analyzed by real-time PCR. Among those 15 genes, the mRNA abundance of 1 gene was found to be significantly correlated with egg quality. This clone (PHB2) exhibited significant sequence similarity with rat prohibitin 2 (Table 3). Its mRNA abundance in the eggs was negatively correlated (R = -0.47, p < 0.05) with the percentage normal alevins at yolk-sac resorption. In addition the mRNA abundance of this gene was significantly higher in eggs exhibiting the lowest developmental potential (Figure 6). An amino acid sequence was generated from nucleotide sequences of Omy.9050 UniGene cluster. This deduced amino acid sequence exhibited 83% identity with zebrafish sequence and 76% identity with human and rat sequences (Figure 6).

## Discussion

### Microarray analysis efficiency and reliability

The hybridization of radiolabeled cDNAs with cDNAs deposited onto nylon membranes has been used for several decades. However, the use of nylon cDNA microarrays is not very common in comparison to glass slide microarrays. Nevertheless, this technology has successfully been used for several years [21]. In our laboratory, we have successfully used this technology to identify differentially expressed genes during oocyte maturation and ovulation [18]. In the present study, we have used the same methodology and have identified a group of 26 genes exhibiting differential egg mRNA abundance after natural controlled ovulation with a false discovery rate of 3.4%. Using real-time PCR, the egg mRNA abundance of 17 genes was analyzed. Among those 17 genes, only 2 exhibited expression patterns totally inconsistent with microarray data. In contrast, the expression patterns of the other genes were very similar to microarray data, even though observed differences were not always significant. It is noteworthy that the 2 genes exhibiting inconsistent expression patterns between PCR and microarray experiments correspond to uncharacterized proteins. Indeed, one of the genes (CA388269) had no significant hit in the Swiss-Prot database while the other one (BX082249) had a significant hit with a hypothetical yeast protein (Table 1). To conclude, the overall consistency of PCR and microarray data suggests that the microarray analysis performed in the present study is robust and reliable.

### Genes exhibiting a differential mRNA abundance after natural or controlled ovulation

#### Hormonal induction of ovulation

Among identified genes, APOC1 and HCK were the most affected by a hormonal-induction of ovulation. Thus, the egg mRNA abundance of those 2 genes was dramatically increased after hormonal induction of ovulation in comparison to natural ovulation (Figure 4). Human APOCs are protein constituents of chylomicrons, very low density lipoproteins, and high-density lipoproteins [22]. The human APOC1 protein is predominantly expressed in liver and adipose tissue [23]. APOC1 may modulate the activity of plasma enzymes involved in lipid metabolism. Besides, APOC1 has also been reported to interfere with the APOE-dependent hepatic uptake of lipoprotein remnants by the low density lipoprotein receptor (LDLr) and LDLr-related protein [24]. Interestingly, it was previously shown in rainbow trout that the same clone of the APOC1 gene was significantly up-regulated in the ovary at the time of oocyte maturation [18]. This could be related to the arrest of lipoproteins uptake by the oocyte at the end of vitellogenesis concomitantly with a decrease of the expression of vitellogenin receptor [25]. It is therefore possible that the hormonal induction of ovulation induces an artificial over abundance of some hormonally-dependent genes, such as APOC1, in the eggs. However, the possible consequences of such an over abundance on lipid metabolism of the embryo is so far unknown.

Similarly to APOC1, the egg mRNA abundance of HCK gene was also dramatically increased after hormonal induction of ovulation. HCK, hemopoietic cell kinase, belongs to Src-familly tyrosine kinases and is expressed in cells of myelomonocytic lineage, B lymphocytes, and embryonic stem cells. It was previously shown that the conventional progesterone receptor could interact, in a progestin-dependent manner, with various signaling molecules, including Src tyrosine kinases [26]. Indeed, these authors used downregulated HCK as a general model of the c-Src family tyrosine kinases to investigate the mechanism of activation by conventional progesterone receptor. In addition, the participation of the conventional progesterone receptor in African clawed frog (*Xenopus laevis*) oocyte maturation process was seriously suggested by two independent studies [27,28]. Besides, Src tyrosine kinase activation has been shown to be one of the earliest transcription-independent responses of Xenopus oocytes to progesterone during in vitro induced maturation; a period when oocyte mRNA content remains stable [29]. Interestingly, we observed a dramatic over abundance of HCK mRNA in the eggs after hormonal induction of ovulation. To date, the significance of this over abundance as a result of hormonally-induced ovulation is unknown. However, it further demonstrates that the egg mRNA abundance of specific genes can be dramatically affected by a hormonal induction of ovulation.

In addition to APOC1 and HCK, eggs obtained after hormonal induction of ovulation were also characterized by higher NTAN1 and lower MR-1 mRNA abundance. However, the fold difference observed for those 2 genes was less important. In mice it has been shown that NTAN1 encodes an N-terminal amidohydrolase specific for N-terminal asparagines, which is involved in ubiquitin-proteasome proteolysis termed as the N-end rule pathway [30]. N-end rule pathway determines metabolic instability of different proteins that contain a destabilizing N-terminal residue [31]. More specifically, a recent study suggested that an over expression of NTAN1 using recombinant NTAN1 adenovirus vector resulted in a marked decrease in the microtubule-associated protein 2 (MAP2) expression in hippocampal neurons in rat [32]. Regardless of the specific target of NTAN1 in the oocyte, an increased expression of this enzyme should participate in protein turnover, and its regulation might be important for the normal development of the oocyte. The second gene, MR-1, is a newly identified protein that interacts with contractile proteins and exists in human myocardial myofibrils [33].

Finally, the egg mRNA abundance of RPL24 was higher after hormonal induction of ovulation. However, this difference was only significant in comparison with the PM group. The 60S ribosomal protein L24 (RPL24) is one of the forty seven 60S ribosomal proteins present in eukaryotic organisms and often used as markers for phylogenetic studies and comparative genomics. Those ribosomal proteins have been sequenced recently in catfish (*Ictalurus punctarus*) and high similarities with mammalian ribosomal protein were found [34]. 60S ribosomal subunit participates in translational initiation in combination with 40S ribosomal subunit [35]. An insertional mutagenesis study carried out in zebrafish (*Danio rerio*) reported this gene to be essential for early embryonic development. Mutation of this gene resulted in small head/eyes mutants [36]. Interestingly, when monitoring embryonic development in the present study, we noticed that many embryos originating from eggs of hormonally-induced females exhibited small eyes at eyeing stage. Precise quantification of this phenomenon would be necessary to stress its relationship with RPL24 over abundance in the eggs.

#### Photoperiodic control of ovulation

Four genes exhibited differential egg mRNA abundance after photoperiod treatment in comparison to natural ovulation. Similarly to eggs obtained after hormonal induction of ovulation, eggs of the PM group also exhibited increased levels of APOC1 and HCK. The differential abundance of both genes was high but less pronounced than after hormonally-induced ovulation. In addition eggs obtained after photoperiod manipulation of ovulation were also characterized by higher MYO1B and PYC mRNA abundance. According to the gene ontology analysis, MYO1B is a cytoskeleton protein involved in nervous system development (Table 2). It is also expressed in a wide variety of tissues including rat neonatal tissues [37,38]. The class I myosin, MYO1B, is a calmodulin- and actin-associated molecular motor widely expressed in mammalian tissues [39]. MYO1B can interact on the dynamic actin filament populations and might play a role in intracellular membrane trafficking [40]. Myosin light chain has been recently suggested to participate in anchoring the 26S proteasome, a 26S multiprotein complex that catalyses the breakdown of polyubiquitylated proteins, to the actin cytoskeleton of goldfish oocyte [41]. Degradation of proteins mediated by ubiquitin-proteasome pathway plays important roles in the regulation of eukaryotic cell cycle [42] and can be involved in oocyte maturation and further embryonic cell cleavages.

Pyruvate carboxylase (PYC) is a mitochondrial biotin-dependent carboxylase. In the adipose tissue and liver PYC participates in the citrate shuttle by which NADPH equivalents are transported out of mitochondria to the cytosol for lipogenesis [43]. Five alternative forms of rat pyruvate carboxylase cDNAs have been identified in liver, kidney, brain, and adipose tissue and these are expressed in a tissue-specific manner [44-46]. In red Seabream (*Pagrus major*), PYC mRNA was detected by Northen blot analysis in heart, liver, muscle and ovary [47]. Interestingly, it was previously shown that a photoperiod manipulation of spawning date was associated with a significantly higher occurrence of yolk-sac resorption defects [48]. Together, these observations suggest a putative link between an abnormal stockpiling of PYC mRNA in the egg and problems in the processing and/or use of yolk-sac lipidic stores. Indeed, it was previously reported that non viable gilthead sea bream eggs have lower pyruvate carboxylase activity than viable eggs [49].

### Genes exhibited an egg mRNA abundance correlated with egg's developmental potential

From microarray data, 30 genes were identified as exhibiting an egg mRNA abundance correlated with egg's developmental potential. However, the false discovery rate was elevated and those genes were considered as candidate genes requiring PCR validation. Nevertheless, it is noteworthy that the ontological analysis of this group showed that 5 genes are involved in the regulation of transcription and others in cell proliferation/development and cytoskeleton organization and biogenesis. In addition, the correlation was confirmed for 1 of the 15 genes analyzed by real-time PCR: prohibitin 2 (PHB2). In animals and yeast, prohibitins have been shown to play important roles in cell cycling and senescence. One of prohibitin 2 major role is to be a chaperone-like regulator of the AAA protease in the mitochondrial matrix that assists in the assembly of inner membrane complex [50]. In *Caenorhabditis elegans*, PHB proteins were showed to be essential during embryonic development and are required for somatic and germ line differentiation in the larval gonad [51]. Moreover, deletions of the *Saccharomyces cerevisiae *homologues, PHB1 and PHB2, result in a decreased replicative lifespan, and a defect in mitochondrial membrane potential. The prohibitin protein has been immunolocalized in mammalian oocytes and embryos and suggested to have an antiproliferative activity [52]. Besides, a higher immunoreactivity level was found in the nucleus of embryo that failed to develop normally in comparison to morphologically normal ones. In the present study, we observed a higher prohibitin 2 mRNA abundance in eggs exhibiting the lowest developmental potential. This differential abundance in eggs of varying quality suggests that prohibitin 2 plays a role in the developmental potential of the embryo. Further studies are needed to unravel the link between an overabundance of prohibitin 2 mRNA in the eggs and a reduced egg developmental potential. Thus, this overabundance could be the result of a reduced prohibitin 2 synthesis during oogenesis.

## Conclusion

In the present study we successfully used rainbow trout cDNA microarrays to analyze egg transcriptome after natural and controlled ovulation and in relationship with the developmental potential of the eggs. We showed that the control of ovulation using either a hormonal induction or a manipulated photoperiod could induce differences in the egg mRNA abundance of specific genes.

In addition, we showed that the egg mRNA abundance of prohibitin 2 (PHB2) was negatively correlated with the developmental potential of the egg.

Furthermore, the identity and ontology of identified genes provided significant hints that could result in a better understanding of the mechanisms associated with each type of ovulation control (e.g hormonal, photoperiodic) or conserved mechanisms triggering a loss of egg developmental potential.

## Methods

### Animals

Investigations were conducted according to the guiding principles for the use and care of laboratory animals and in compliance with French and European regulations on animal welfare. Three groups of male and female rainbow trout (*Oncorhynchus mykiss*) were obtained from our experimental fish farm (Sizun, France) and maintained until reproductive season under natural photoperiod and water temperature conditions. A first set of egg samples was collected from females undergoing natural (N) ovulation. Four weeks before expected ovulation fish (25 females) were transferred in a controlled recirculated water system (12°C) under natural photoperiod in INRA experimental facilities (Rennes, France). A second set of egg samples was collected from females subjected to a hormonal induction (HI) of ovulation. Four weeks before expected ovulation fish were transferred in a controlled recirculated water system (12°C) under natural photoperiod in INRA experimental facilities (Rennes, France). Females (n = 33) were given a 250 μL.Kg^-1 ^body weight (b.w) intraperitoneal injection of [Des-Gly^10^, DArg^6^, Pro-NHEt^9^]-GnRH analog (Bachem, Allemagne) at 60 μg.Kg^-1 ^b.w. A third set of egg samples was collected from females subjected to a photoperiod manipulation (PM) of ovulation. After a first reproduction, fish (17 females) were isolated in light-proofed tanks and exposed to an artificial photoperiod. Beginning on January 15^th^, all fish were held under constant light (24L:0D) for 490°C.day. Then, beginning on March 27^th^, they were held under short photoperiod (8L:16D) until ovulation (1230°C.day). Light was supplied by 4 neon tubes (58 Watts).

### Gamete collection

In order to avoid excessive post-ovulatory ageing, unfertilized eggs were collected by manual stripping 5 days after detected ovulation. Two batches of 5 mL of eggs (approximately 100 to 200 eggs per batch) were used for fertilization. At each egg collection day, fresh sperm samples were collected from 10 mature males originating from the same group in order to fertilize eggs with a pool of sperms. Sperm samples were obtained by manual pressure on the abdomen and kept at 4°C for a short time before use.

### Fertilization and early development

Fertilization was performed under previously described standardized conditions [16]. The two batches of 5 mL of eggs were fertilized with 5 μl of pooled semen. Fertilized eggs were transferred into compartmentalized incubation trays supplied by recirculated water (10°C). Water temperature and chemistry were routinely monitored and maintained constant over the entire incubation period. Dead eggs and embryos were periodically removed and survival rates were estimated as percentages of the initial number of eggs used for fertilization. Survival at the completion of yolk sac resorption (YSR, 550°C.day) was monitored. The occurrence of noticeable morphological malformations at YSR was also monitored. Survival and malformation data were used to calculate the proportion of normal alevins at YSR expressed as a percentage of the initial number of eggs.

### RNA extraction

Extractions were performed as previously described [53] with minor modifications. Total RNA was extracted from 20 unfertilized eggs using 9 mL of TRizol (Invitrogen) in 13 mL sterile polypropylene tubes. Because of high egg vitellogenic content, each RNA was subsequently repurified using a Nucleospin RNA 2 kit (Macherey Nagel) in order to obtain genomic-grade RNA quality. For each egg sample, three RNA extracts were obtained, pooled and precipitated with sodium acetate (3 M, pH5.2, Prolabo) to increase RNA concentration. Thus, any RNA sample used for transcriptomic analysis originated from 60 unfertilized eggs of an individual female.

### cDNA microarrays

Nylon micro-arrays (7.6 × 2.6 cm) were obtained from INRA-GADIE (Jouy-en-Josas, France) resource center [54]. A set of 9152 distinct rainbow trout cDNA clones originating from 2 pooled-tissues library [55,56] were spotted in duplicates after PCR amplification. PCR products were spotted onto Hybond N+ membranes as previously described [57]. This rainbow trout generic array was deposited in Gene Expression Omnibus (GEO) database (Platform# GPL3650) [58].

### Microarray hybridization

Four RNA samples originating from naturally ovulating females, 11 RNA samples originating from hormonally-induced females and 14 RNA samples originating from photoperiod-manipulated females were used for microarray hybridization according to the following procedure. Hybridizations were carried out as previously described [21], with minor modifications, at INRA genomic facility (Rennes). A first hybridization was performed using a 33P-labelled oligonucleotide (TAATACGACTCACTATAGGG which is present at the extremity of each PCR product) to monitor the amount of cDNA in each spot. After stripping (3 hours 68°c, 0.1× SSC, 0.2% SDS), arrays were prehybridized for 1 h at 65°C in hybridization solution (5× Denhardt's, 5× SSC, 0.5% SDS). Complex probes were prepared from 3 μg of RNA by simultaneous reverse transcription and labelling for 1 hour at 42°C in the presence of 50 μCi [alpha-33P] dCTP, 5 μM dCTP, 0.8 mM each dATP, dTTP, dGTP and 200 units M-MLV SuperScript RNase H-reverse transcriptase (GIBCO BRL) in 30 μL final volume. RNA was degraded by treatment at 68°C for 30 min with 1 μl 10% SDS, 1 μl 0.5 M EDTA and 3 μl 3 M NaOH, and then equilibrated at room temperature for 15 min. Neutralization was done by adding 10 μl 1 M Tris-HCl plus 3 μl 2N HCl. Arrays were incubated with the corresponding denatured labeled cDNAs for 18 h at 65°C in hybridization solution. After 3 washes (1 hours 68°C, 0.1× SSC 0.2% SDS), arrays were exposed 65 hours to phosphor-imaging plates before scanning using a FUJI BAS 5000. Signal intensities were quantified using ArrayGauge software (FujifilmMedical Systems, Stanford, CT) and deposited in GEO database (Series# GSE5928) [58].

### Microarray signal processing

Spots with low oligonucleotide signal (lower than three times the background level) were excluded from the analysis. After this filtering step, signal processing was performed using the vector oligonucleotide data to correct each spot signal by the actual amount of DNA present in each spot. After correction, signal was normalized by dividing each gene expression value by the median value of the array.

### Microarray data analysis

Statistical analysis was performed using Significance Analysis of Microarray (SAM) software [59]. For each comparison, the lowest false discovery rate (FDR) was used to identify differentially abundant genes. A first analysis was performed in order to identify differentially abundant transcripts between N group and the two other experimental groups (HI and PM). A second analysis was performed in order to identify differentially abundant transcripts in relation with egg quality, estimated by percentage of normal alevins at YSR within the complete data set or inside each experimental group (HI and PM).

### Identity of mircroarray cDNA clones

Rainbow trout sequences originating from INRA AGENAE [55] and USDA [56] EST sequencing programs were used to generate publicly available contigs [60]. The 8th version (Om.8, released January 2006) was used for BlastX [61] comparison against the Swiss-Prot database (January 2006) [62]. The score of each alignment was retrieved after performing BlastX comparison. This was performed automatically for each EST spotted onto the membrane and used to annotate the 9152 clones of the microarray.

### Data mining

For all the clones identified as differentially abundant after a SAM analysis (Table 1, 3) the official human gene symbol was retrieved [63] and used in the text, figures and tables for clarity reasons. In addition, the accession number of the corresponding rainbow trout cluster (UniGene Trout, January 2006), if any, was retrieved from the UniGene database [64]. For all genes identified as differentially abundant in the transcriptomic analysis, ontologies were obtained using the AmiGO tool [65]. Finally, for the differentially abundant genes identified in the real-time PCR analysis, a BlastX search was performed against the GenBank NR database. When possible, this was done using the contig sequence generated from all the ESTs present in the corresponding UniGene cluster. Subsequently, the amino acid sequence deduced from the trout contig sequence was aligned with cognate vertebrate forms.

### Real-time PCR analysis

Real-time PCR was performed using all RNA samples used for microarray analysis (N = 29). Reverse transcription and real time PCR were performed as previously described [66]. Briefly, 2 μg of total RNA were reverse transcribed using 200 units of Moloney murine Leukemia virus (MMLV) reverse transcriptase (Promega, Madison, WI) and 0.5 μg dT15 Oligonucleotide (Promega) per μg of total RNA according to manufacturer's instruction. RNA and dNTPs were denatured for 6 min at 70°C; then chilled on ice for 5 min before the reverse transcription master mix was added. Reverse transcription was performed at 37°C for 1 hour and 15 min followed by a 15 min incubation step at 70°C. Control reactions were run without MMLV reverse transcriptase and used as negative controls in the real-time PCR study. Real-time PCR experiments were conducted using an I-Cycler IQ (Biorad, Hercules, CA). Reverse transcription products were diluted to 1/25, and 5 μl were used for each real-time PCR reaction. Triplicates were run for each RT product. Real-time PCR was performed using a real-time PCR kit provided with a SYBR Green fluorophore (Eurogentec, Belgium) according to the manufacturer's instructions and using 600 nM of each primer. After a 2 min incubation step at 50°C and a 10 min incubation step at 95°C, the amplification was performed using the following cycle: 95°C, 20 sec; 60°C, 1 min, 40 times. The relative abundance of target cDNA within sample set was calculated from a serially diluted oocyte cDNA pool using the I-Cycler IQ software. After amplification, a fusion curve was obtained using the following protocol: 10 sec holding followed by a 0.5°C increase, repeated 80 times and starting at 55°C. The level of CyclinA2 RNAs was monitored using the same sample set to allow normalization. Cyclin A2 was used for normalization because its mRNA abundance was shown to be elevated and highly stable in rainbow trout eggs collected 5 days after ovulation ([16]). Statistical analyses were performed using Statistica 7.0 software (Statsoft, Tulsa, OK). Differences between groups were analyzed using non parametric U tests.

## Authors' contributions

EB performed egg quality monitoring, real-time PCR study, microarray analysis and drafted the manuscript. AF and JB coordinated the study and participated in real-time PCR and microarray data analysis, and in manuscript writing. All authors read and approved the final manuscript.

## Figures and Tables

**Table 1:** Genes exhibiting differential egg mRNA abundance among experimental groups identified from the microarray analysis.

**Clones**	**GenBank**	**Sigenae contig**	**Symbol**	**swissprot_hit_description**	**Score**	**UniGene**
**tcac0001.c.18**	BX082249	**tcac0001c.c.18_5.1.s.om.8**		**YEAST (P53230) Hypothetical 44.2 kDa protein in RME1-TFC4 intergenic region**	**283**	
**1RT159P21_B_H11**	CA388269	**CA388269.1.s.om.8**				
tcbk0051.e.02	BX878405	tcay0028b.c.19_3.1.s.om.8				
**1RT65F10_D_C05**	CA353171	**tcab0001c.m.15_5.1.s.om.8**	**APOC1**	**MOUSE (P34928) Apolipoprotein C-I precursor (Apo-CI) (ApoC-I)**	**123**	**Omy.10219**
1RT121B08_D_A04	CA359367	CA359367.1.s.om.8	CTNNBL1	HUMAN (Q8WYA6) Beta-catenin-like protein 1 (Nuclear-associated protein)	950	Omy.23137
**1RT56O04_C_H02**	CA351228	**CA351228.1.s.om.8**	**DAB2**	**MOUSE (P98078) Disabled homolog 2 (DOC-2)**	**215**	
**1RT64F24_D_C12**	CA358202	**CA358202.1.s.om.8**	**DBNL**	**MOUSE (Q62418) Drebrin-like protein (SH3 domain-containing protein 7)**	**720**	
1RT87E10_C_C05	CA345343	tcay0028b.g.03_3.1.s.om.8	DDAH2	MOUSE (Q99LD8) NG, NG-dimethylarginine dimethylaminohydrolase 2	629	Omy.23405
**1RT68D18_D_B09**	CA34327	**tcba0017c.p.21_5.1.s.om.8**	**HCK**	**MOUSE (P08103) Tyrosine-protein kinase HCK (EC 2.7.1.112)**	**324**	**Omy.9448**
**tcay0037.m.11**	BX319623	**tcay0032b.l.02_3.1.s.om.8**	**HNRPK**	**RAT (P61980) Heterogeneous nuclear ribonucleoprotein K**	**1171**	**Omy.26818**
tcay0030.n.02	BX316222	tcav0005c.k.03_3.1.s.om.8	HSPA9B	HUMAN (P38646) Stress-70 protein, mitochondrial precursor	901	Omy.26983
1RT162C23_A_B12	CA382140	tcbk0012c.o.01_5.1.s.om.8	ING1	HUMAN (Q9UK53) Inhibitor of growth protein 1	435	Omy.24666
**1RT121G15_A_D08**	CA362639	**tcay0007b.n.06_3.1.s.om.8**	**LYPA3**	**HUMAN (Q8NCC3) 1-O-acylceramide synthase precursor (EC 2.3.1.-)**	**816**	**Omy.9525**
**tcbk0023.o.24**	BX875550	**tcbk0005c.o.10_5.1.s.om.8**	**MR-1**	**ARATH (O24496) Hydroxyacylglutathione hydrolase cytoplasmic (EC 3.1.2.6)**	**466**	**Omy.19659**
**1RT131K20_C_F10**	CA383630	**CA383630.1.s.om.8**	**MYO1B**	**MOUSE (P46735) Myosin Ib (Myosin I alpha) (MMI-alpha) (MMIa)**	**985**	
**tcba0025.n.15**	BX866389	**tcay0008b.e.10_3.1.s.om.8**	**NTAN1**	**HUMAN (Q96AB6) Protein N-terminal asparagine amidohydrolase (EC 3.5.1)**	**510**	
tcbk0049.m.03	BX884905	tcbk0002c.c.19_5.1.s.om.8	OSBPL5	MOUSE (Q9ER64) Oxysterol binding protein-related protein 5	344	Omy.14649
**tcbk0050.a.20**	BX886190	**tcbk0050c.a.20_5.1.s.om.8**	**PGH2**	**CHICK (P27607) Prostaglandin G/H synthase 2 precursor**	**1636**	**Omy.20943**
**tcbk0055.m.20**	BX880138	**tcbk0055c.m.20_5.1.s.om.8**	**PKP1**	**HUMAN Plakophilin 1**	**337**	
**tcay0027.b.13**	BX313624	**tcay0027b.b.13_5.1.s.om.8**	**PYC**	**HUMAN (P11498) Pyruvate carboxylase, mitochondrial precursor (EC 6.4.1.1)**	**1021**	
**1RT67N13_B_G07**	CA360456	**CA355135.1.s.om.8**	**RBM5**	**HUMAN (P52756) RNA-binding protein 5 (Putative tumor suppressor LUCA15)**	**296**	
tcbk0027.b.05	BX887647	tcbi0025c.k.02_5.1.s.om.8	RL10	HUMAN (P27635) 60S ribosomal protein L10	1039	Omy.4144
**1RT139F11_B_C06**	CA384643	**tcay0034b.h.11_3.1.s.om.8**	**RPL24**	**GILMI (Q9DFQ7) 60S ribosomal protein L24**	**529**	**Omy.9444**
1RT63M02_C_G01	CA343028	tcac0004c.h.08_5.1.s.om.8	RPN2	HUMAN (P04844) Dolichyl-diphosphooligosaccharide-protein glycosyltransferase 63 kDa subunit precursor (Ribophorin II)	643	Omy.24414
**tcad0007.p.12**	BX078856	**tcac0005c.e.12_3.1.s.om.8**	**SEC22**	**YARLI (Q6C880) Protein transport protein SEC22**	**237**	**Omy.913**
1RT56L15_B_F08	CA351681	CA351681.1.s.om.8	TPH	XENLA (Q92142) Tryptophan 5-hydroxylase (EC 1.14.16.4)	725	

Genes subsequently studied by real time PCR are bolded. For each gene, clone name, GenBank accession number, official human symbol and corresponding UniGene cluster are indicated. The Sigenae contig name [60] used for Blast comparison against the Swiss-Prot database is shown. Resulting best hit and corresponding score are indicated.

**Table 2:** Ontologies of the genes exhibiting differential egg mRNA abundance among experimental groups identified from the microarray analysis.

**Symbol**	**Biological Process (P)**	**Cellular component (C)**	**Molecular Function (F)**
APOC1	negative regulation of lipoprotein lipase activity negative regulation of binding lipid metabolism	chylomicron	enzyme activator activity lipid binding
CTNNBL1	induction of apoptosis	nucleus	
DAB2	cell proliferation		protein binding
DBNL	Rac protein signal transduction activation of JNK activity	Lamellipodium Cytoplasm cell cortex	actin binding enzyme activator activity protein binding
DDAH2	anti-apoptosis arginine catabolism nitric oxide mediated signal transduction		hydrolase activity
HCK	protein amino acid phosphorylation mesoderm development		protein-tyrosine kinase activity protein binding
HNRPK		nucleus	protein binding single-stranded DNA binding
HSPA9B	hemopoiesis	mitochondrial matrix	ATPase activity ATP binding
ING1	negative regulation of cell proliferation negative regulation of cell growth	nucleus	DNA binding
LYPA3	fatty acid catabolism	lysosome	phospholipid binding lysophospholipase activity
MR-1			
MYO1B	nervous system development	Cytoskeleton brush border	motor activity
NTAN1	memory adult locomotory behavior	Nucleus cytoplasm	protein N-terminal asparagine amidohydrolase activity
OSBPL5	cholesterol metabolism cholesterol transport Golgi to plasma membrane transport	integral to membrane cytosol	oxysterol binding
PGH2	physiological process keratinocyte differentiation cyclooxygenase pathway	Nucleus cytoplasm	Peroxidase activity prostaglandin-endoperoxide synthase activity
PKP1	signal transduction cell adhesion	desmosome nucleus intermediate filament	structural constituent of epidermis signal transducer activity intermediate filament binding
PYC			ATP binding biotin binding pyruvate carboxylase activity
RBM5	RNA binding DNA binding	nucleus	RNA processing
RPL10	Spermatogenesis protein biosynthesis	cytosolic large ribosomal subunit (sensu Eukaryota) mitochondrial large ribosomal subunit	structural constituent of ribosome
RPL24	translation	mitochondrial large ribosomal subunit	structural constituent of ribosome
RPN2	protein modification protein amino acid N-linked glycosylation	oligosaccharyl transferase complex	
SEC22	ER to Golgi vesicle-mediated transport	endoplasmic reticulum membrane	transporter activity
TPH1	serotonin biosynthesis from tryptophan	cytoplasm	tryptophan 5-monooxygenase activity
YG1W	protein import into mitochondrial matrix	mitochondrion	Protein binding

**Table 3:** Genes exhibiting differential mRNA abundance in eggs of varying quality identified from the microarray analysis

**Symbol**	**Genbank**	**Sigenae contig**	**Symbol**	**swissprot_hit_description**	**Score**	**UniGene**
tcbr0001.b.08		NO CONTIG				
**1RT42N11_B_G06**	CA378261	**tcba0024c.d.03_5.1.s.om.8**	**AGM1**	**HUMAN (O95394) Phosphoacetylglucosamine mutase (EC 5.4.2.3)**	**1065**	**Omy.22147**
1RT120K08_C_F04	CA362248	CA362248.1.s.om.8	ALG2	HUMAN (Q9H553) Alpha-1,3-mannosyltransferase ALG2 (EC 2.4.1.-)	662	
1RT85M16_C_G08	CA345100	tcac0001c.c.13_3.1.s.om.8	APOB	HUMAN (P04114) Apolipoprotein B-100 precursor	630	Omy.8599
**tcay0008.m.21**	BX301016	**tcav0001c.l.14_3.1.s.om.8**	**BMP7**	**MOUSE (P23359) Bone morphogenetic protein 7 precursor (BMP-7)**	**1154**	**Omy.19556**
**tcbk0001.p.13**	BX873334	**tcbk0001c.p.13_5.1.s.om.8**	**CASZ1**	**HUMAN (Q86V15) Probable transcription factor CST**	**234**	**Omy.20281**
**tcay0007.f.20**	BX300279	**tcay0007b.f.20_5.1.s.om.8**	**CF188**	**RAT (Q5FWS4) Protein C6orf188 homolog**	**468**	**Omy.26998**
**tcba0030.i.17**	BX867113	**tcay0040b.g.08_5.1.s.om.8**	**CUL5**	**RABIT (Q29425) Cullin-5 (CUL-5)**	**1694**	**Omy.21358**
**1RT63D22_D_B11**	CA343059	**CA343059.1.s.om.8**	**DCPS**	**MOUSE (Q9DAR7) Scavenger mRNA decapping enzyme DcpS**	**195**	
tcba0010.c.10	BX861936	tcba0010c.c.10_5.1.s.om.8	DUSP24	HUMAN (Q9Y6J8) Dual specificity protein phosphatase 24	688	
1RT104M18_C_G09	CA347317	tcay0009b.d.09_3.1.s.om.8	FGD5	MOUSE (Q80UZ0) FYVE, RhoGEF and PH domain containing protein 5	145	Omy.10646
**1RT147H01_B_D01**	CA350285	**tcad0009a.n.22_3.1.s.om.8**	**GMCL1**	**HUMAN (Q96IK5) Germ cell-less protein-like 1**	**1353**	**Omy.2306**
tcbk0042.i.19	BX879311	tcav0003c.a.13_3.1.s.om.8	GTF2B	RAT (P62916) Transcription initiation factor IIB	1449	
**tcay0006.d.12**	BX299916	**tcab0003c.m.12_5.1.s.om.8**	**HCFC1**	**MOUSE (Q61191) Host cell factor (HCF) (HCF-1)**	**695**	
**tcay0009.k.03**	BX302686	**tcay0009b.k.03_5.1.s.om.8**	**KHK**	**HUMAN (P50053) Ketohexokinase (EC 2.7.1.3)**	**591**	
1RT68H23_B_D12	CA343227	CA343227.1.s.om.8	KIF4A	XENLA (Q91784) Chromosome-associated kinesin KLP1	477	
tcbk0045.a.13	BX883207	tcbk0045c.a.13_5.1.s.om.8	LAMB2	HUMAN (P55268) Laminin beta-2 chain precursor (S-laminin)	1097	
1RT77F09_B_C05	CA354296	CA354296.1.s.om.8	LRTM1	PONPY (Q5R6B1) Leucine-rich repeat transmembrane neuronal protein 1 precursor	410	
**tcbk0030.p.11**	BX885788	**tcbk0030c.p.11_5.1.s.om.8**	**MCF2L**	**MOUSE (Q64096) Guanine nucleotide exchange factor DBS**	**468**	**Omy.6690**
1RT79A16_C_A08	CA355005	CA355005.1.s.om.8	NEK1	MOUSE (P51954) Serine/threonine-protein kinase Nek1 (EC 2.7.1.37)	359	
1RT94E22_C_C11	CA347857	CA347857.1.s.om.8	OBSCN	CAEEL (O01761) Muscle M-line assembly protein unc-89	271	Omy.24205
tcab0002.l.03	BX080933	tcab0002c.l.03_5.1.s.om.8	PDCL3	HUMAN (Q9H2J4) Phosducin-like protein 3	305	
tcbk0044.f.24	BX877936	tcbk0044c.f.24_5.1.s.om.8	PDGFRA	XENLA (P26619) Alpha platelet-derived growth factor receptor precursor	248	
**tcay0023.m.15**	BX310740	**tcay0002b.p.08_3.1.s.om.8**	**PHB2**	**RAT (Q5XIH7) Prohibitin-2 (B-cell receptor-associated protein BAP37) (BAP-37)**	**1013**	**Omy.9050**
**tcay0029.o.17**	BX314382	**tcay0029b.o.17_3.1.s.om.8**	**RAB3IP**	**HUMAN (Q96QF0) RAB3A-interacting protein (Rabin-3)**	**206**	
1RT149L18_D_F09	CA350883	tcay0003b.j.14_3.1.s.om.8	TF	SALSA (P80426) Serotransferrin I precursor (Siderophilin I) (STF I)	663	Omy.9801
1RT142A22_C_A11	CA349568	CA349568.1.s.om.8	TGFBR2	RAT (P38438) TGF-beta receptor type II precursor (EC 2.7.1.37)	1133	Omy.23150
**tcay0018.b.11**	BX307666	**tcab0003c.i.10_5.1.s.om.8**	**TLE1**	**MOUSE (Q62440) Transducin-like enhancer protein 1 (Groucho-related protein 1)**	**2032**	**Omy.9672**
1RT165F23_B_C12	CA388009	CA388009.1.s.om.8	VWF	HUMAN (P04275) Von Willebrand factor precursor (vWF	579	
**tcay0018.a.17**	BX307636	**tcay0009b.k.21_3.1.s.om.8**	**ZNF16**	**HUMAN (P17020) Zinc finger protein 16 (Zinc finger protein KOX9)**	**311**	**Omy.6191**
**tcbk0009.g.08**	BX875276	**tcbk0009c.g.08_5.1.s.om.8**	**ZNF261**	**MOUSE (Q9JLM4) Zinc finger protein 261 (DXHXS6673E protein)**	**254**	**Omy.2759**

Genes subsequently studied by real time PCR are bolded. For each gene, clone name, GenBank accession number, official human symbol and corresponding UniGene cluster are indicated. The Sigenae contig name [60] used for Blast comparison against the Swiss-Prot database is shown. Resulting best hit and corresponding score are indicated.

**Table 4:** Ontologies of the genes exhibiting differential mRNA abundance in eggs of varying quality identified from the microarray analysis.

**Symbol**	**Biological Process (P)**	**Cellular component (C)**	**Molecular Function (F)**
AGM1	glucosamine metabolism		phosphoacetylglucosamine mutase activity
APOB	circulation lipid transport signal transduction	extracellular region endoplasmic reticulum microsome	lipid transport activity receptor binding
BMP7	BMP signalling pathway cell development organ morphogenesis mesoderm formation pattern specification positive regulation of cell differentiation	Extracellular space	cytokine activity protein binding
CUL5	cell cycle arrest cell proliferation G1/S transition of mitotic cell cycle induction of apoptosis by intracellular signals negative regulation of cell proliferation regulation of progression through cell cycle		calcium channel activity protein binding receptor activity
DCPS	mRNA catabolism		pyrophosphatase activity
FGD5	cytoskeleton organization and biogenesis regulation of cell shape	cytoplasm Golgi apparatus	protein binding small GTPase binding
GMCL1	nuclear membrane organization and biogenesis regulation of transcription spermatogenesis	nuclear lamina nuclear matrix	protein binding
GTF2B	mRNA transcription from RNA polymerase II promoter transcription initiation from RNA polymerase II promoter	transcription factor complex	general RNA polymerase II transcription factor activity protein binding RNA polymerase II transcription factor activity
HCFC1	positive regulation of progression through cell cycle regulation of transcription transcription from RNA polymerase II promoter	cytoplasm nucleus	identical protein binding transcription coactivator activity transcription factor activity
KHK	carbohydrate catabolism		ketohexokinase activity
KIF4A	organelle organization and biogenesis anterograde axon cargo transport	cytoplasm spindle microtubules	microtubule motor activity
LAMB2	synaptic transmission electron transport	basal lamina membrane	calcium ion binding oxidoreductase activity phospholipase A2 activity structural molecule activity
MCF2L	Rho protein signal transduction	membrane lamellipodium	phosphatidylinositol binding Rho guanyl-nucleotide exchange factor activity
NEK1	response to DNA damage stimulus response to ionizing radiation	cytoplasm nucleus	protein binding protein kinase activity
PDCL3	phototransduction	cytoplasm	protein binding
PDGFRA	cell proliferation extracellular matrix organization and biogenesis male genitalia development morphogenesis	integral to plasma membrane	platelet-derived growth factor binding protein dimerization activity protein serine/threonine kinase activity
PHB2	signal transduction negative regulation of transcription	mitochondrial inner membrane nucleus	estrogen receptor binding protein binding specific transcriptional repressor activity
RAB3IP		cytoplasm nucleus	protein Binding GTPase binding
TGFBR2	regulation of cell proliferation Cell fate commitment protein amino acid phosphorylation protein amino acid dephosphorylation	integral to membrane	ATP binding protein binding protein tyrosine kinase activity
TLE1	signal transduction regulation of transcription, DnA-dependent organ morphogenesis	nucleus	
VWF	cell adhesion response to wounding	extracellular space	protease binding protein binding

**Table 5:** Real-time PCR primer sequences. For each target gene, official symbol of the human protein, GenBank accession number of the clone and clone name are indicated

**Reverse sequence**	**Foward sequence**	**Symbol**	**GenBank**	**Clones**
ACTTCCTCCTCCTCCACGTT	CCAGCTCATGTACCCGTTCT		CA388269	1RT159P21_B_H11
CTGGGTTCAGGAAGTGTGGT	TCGCTGGAGGAGTAGGAGAG	AGM1	CA378261	1RT42N11_B_G06
GCTGTCCCCAATCTTTTCAA	GGCTGAGAAGACCATTGAGG	APOC1	CA353171	1RT65F10_D_C05
GTGTCTGGACCGTCTGACCT	CCTGCACAAGTACGTGGAGA	BMP7	BX301016	tcay0008.m.21
GCAGTGGTAGTGGGTCACCT	CTTCCGCCGTTATGATCTGT	CASZ1	BX873334	tcbk0001.p.13
GATCAGTAGCCAACCCAGGA	GCAGTGCCAGGATGAACTTT	CF188	BX300279	tcay0007.f.20
CAACTTGCGTACGATGCTGT	AGGCCTACATTGTGGAGTGG	CUL5	BX867113	tcba0030.i.17
GGAAAGCTGGTTGCTTGCTG	CTTCAAACTCTGCGCCGGCACA	cyclinA2	BX080925	tcab0003.e.11
GAGCTGCTATGGGAGAGGTG	CTAAGGCTGGACGAGGTCTG	DAB2	CA351228	1RT56O04_C_H02
TCCCCTCGTAGGTGAACAAG	CAAGTGTTTGAGCGAACGAA	DBNL	CA358202	1RT64F24_D_C12
TCTCCAACAGGGTGTCTTCC	ACGGAAAGTTGAACGACCAG	DCPS	CA343059	1RT63D22_D_B11
TGGTCTTTCTCCAGGGTGAG	CCTCGGAGGCATCTAGCATA	GMCL1	CA350285	1RT147H01_B_D01
GTGCAAATTTTTGGGGAAGA	CCCGAGATAAGGACTGATGG	HCFC1	BX299916	tcay0006.d.12
GACAAATGATGACAGTGGCCTA	TGCGATGTGATGTGACATTTT	HCK	CA34327	1RT68D18_D_B09
CAGACTTGCCACTGACCAGA	CAGCATCATTGGTGTGAAGG	HNRPK	BX319623	tcay0037.m.11
CCGTGGTCACATTGCTTATG	GGCATGTTGCAGACTTCGTA	KHK	BX302686	tcay0009.k.03
GTTCCCATACGCACATTCCT	TCCCAGCCATCTTCAAAGTC	LYPA3	CA362639	1RT121G15_A_D08
TCGACTCGTACGTCAACTGG	GCTCTCCAACTCTTCGGATG	MCF2L	BX885788	tcbk0030.p.11
CTGTGGTCCCAGTGTTTGTG	GCAGACCCACAGACAGTTCA	MR-1	BX875550	tcbk0023.o.24
CATGGCCAGGATACCATTCT	TTCATCGAACTGACGCTACG	MYO1B	CA383630	1RT131K20_C_F10
AACTAGGTGGCAGGTGGTTG	GAGAGTTTGCAGCCACAACA	NTAN1	BX866389	tcba0025.n.15
TCCTGGATGTGGAAGGAGTC	AAGCTGAAGTTCGACCCAGA	PGH2	BX886190	tcbk0050.a.20
TCGTCCAGGATGATGTTGAA	GTTCAATGCCTCACAGCTCA	PHB2	BX310740	tcay0023.m.15
CAGCAGGGGAGAGATTTCAG	CCAGCCAGAGAGAAGACACC	PKP1	BX880138	tcbk0055.m.20
GAAGGGGATGTTGGTCTTGA	GCATTCCAAGGAGCAGTCAT	PYC	BX313624	tcay0027.b.13
CTCCGGTGTGCCCTAATAAA	GCTGGGCTTCTACCTCACAG	RAB3I	BX314382	tcay0029.o.17
ACGGAGGAGGAAGAGGAGAG	GGGGCAAGGAGAAGAAAGAC	RBM5	CA360456	1RT67N13_B_G07
CAGGCTTCTGGTTCCTCTTG	CAAGAAGGGCCAGTCTGAAG	RPL24	CA384643	1RT139F11_B_C06
AGCTGTGGTGGAGAAGCAAT	GGGGTGGGGGAGATACTAAA	SEC22	BX078856	tcad0007.p.12
TCGTGGGAGATGTCGATACA	GCCAAAGTCTGCTTCTCCTG	TLE3	BX307666	tcay0018.b.11
GAGGAAGGAGGCAGTCACAG	GCTCCACTGGAAGACCATGT	YG1W	BX082249	tcac0001.c.18
GTGCACTCGTAGGGCTTCTC	AACACCTCCGAAGTCACACC	ZNF16	BX307636	tcay0018.a.17
GAGTCCGAGCACTTGGAAAG	AGAGGAGGTGCTGGAGATGA	ZNF261	BX875276	tcbk0009.g.08
